# Cholesterol Induces Nrf-2- and HIF-1*α*-Dependent Hepatocyte Proliferation and Liver Regeneration to Ameliorate Bile Acid Toxicity in Mouse Models of NASH and Fibrosis

**DOI:** 10.1155/2020/5393761

**Published:** 2020-05-25

**Authors:** Yula Kaminsky-Kolesnikov, Einat Rauchbach, Diana Abu-Halaka, Michal Hahn, Carmen García-Ruiz, Jose C. Fernandez-Checa, Zecharia Madar, Oren Tirosh

**Affiliations:** ^1^Institute of Biochemistry, Food Science, And Nutrition, RHS Faculty of Agriculture Food and Environment, The Hebrew University of Jerusalem, Israel; ^2^Department of Cell Death and Proliferation, Institute of Biomedical Research of Barcelona (IIBB), CSIC, Barcelona, Spain; ^3^Liver Unit, Hospital Clinic I, Provincial de Barcelona, IDIBAPS, Barcelona, Spain; ^4^CIBEREHD, Barcelona, Spain; ^5^Research Center for ALPD, Keck School of Medicine, University of Southern California, Los Angeles, CA, USA

## Abstract

Nonalcoholic steatohepatitis (NASH) is currently one of the most common liver diseases worldwide. The toxic effects of lipids and bile acids contribute to NASH. The regenerative pathway in response to damage to the liver includes activation of the inflammatory process and priming of hepatocytes to proliferate to restore tissue homeostasis. However, the effects of cholesterol on bile acid toxicity, inflammation, and fibrosis remain unknown. We have used two mouse models of bile acid toxicity to induce liver inflammation and fibrosis. A three-week study was conducted using wild-type mice receiving an atherogenic diet (1% (*w*/*w*) cholesterol and 0.5% (*w*/*w*) cholic acid) and its separate constituents. Mdr2-/- mice were fed a high-cholesterol-enriched diet or standard AIN-93 diet for 6 weeks. We measured serum transaminase levels to assess liver tissue necrosis and fibrosis; iNOS, SAA1, SAA2, and F4/80 levels to determine liver inflammation; PCNA and HGF levels to evaluate proliferative response; and Nrf-2, HIF-1*α*, and downstream gene expression to establish protective responses. In both studies, high bile acid levels increased serum transaminases and liver fibrosis, whereas cholesterol supplementation attenuated these effects. Cholesterol supplementation activated survival and the robustness of HIF-1*α* and Nrf-2 gene expression in hepatocytes, induced liver inflammation and hepatocyte proliferation, and inhibited stellate cell hyperplasia and fibrosis. In conclusion, our data show for the first time that cholesterol intake protects against bile acid liver toxicity. The balance between hepatic cholesterol and bile acid levels may be of prognostic value in liver disease progression and trajectory.

## 1. Introduction

Nonalcoholic steatohepatitis (NASH) is now one of the most common liver diseases worldwide. The high prevalence of nonalcoholic fatty liver disease (NAFLD) is associated with the increasing global incidence of obesity. The mechanism by which NAFLD progresses to NASH and then to hepatic cirrhosis has not been fully elucidated. It is known, however, that this progression is strongly influenced by the toxic effect of lipids and bile acids. Bile acid toxicity is an important factor in metabolic disorders such as NAFLD and NASH [1]. It also contributes to cholestatic conditions such as primary sclerosing cholangitis and fibropolycystic liver disease [2], cirrhosis and fibrosis [3], biliary stone disease, and cholangiocarcinoma [4].

Cholestasis syndrome is indicative of bile acid toxicity and is detected in 3%, 34%, and 47% of patients with NAFLD, NASH, and liver cirrhosis, respectively. Liver damage was comparatively worse in all forms of NAFLD presenting with cholestasis [5]. Bile acid synthesis, excretion, and reuptake are tightly regulated by the farnesoid X receptor (FXR) [6] and the cholesterol removal pathway. Dysfunctional bile acid absorption by ileal bile acid transporters may result in diarrhea. However, it also ameliorated liver histology in animal models of cholestasis liver disease and NASH [7].

The liver uptake of free cholesterol (FC) also regulates NASH and NAFLD disease progression. Emerging experimental and clinical data have correlated altered hepatic cholesterol homeostasis and accumulation with NASH pathogenesis [8, 9]. NASH is characterized by hepatic steatosis and necroinflammation. The cholesterol-mediated transition towards hepatic inflammation is a key step in NAFLD disease pathogenesis as it may promote liver damage and culminate in hepatic fibrosis, cirrhosis, and liver cancer [10].

When experimental animals receive diets supplemented with cholesterol rather than high-fat diets (HFD) without cholesterol, dietary and liver cholesterol accumulation induces symptoms of NASH resembling those seen in nonobese human subjects with this disorder. These include moderate weight loss, reduction of adipose tissue mass, and little or no hyperinsulinemia [11]. Thus, cholesterol is a nutritional factor critical in the development of liver inflammation [12, 13]. In fact, cholesterol was found to participate in this process [14, 15]. The expression of c-Fos in hepatocytes in response to the accumulation of cholesterol, oxysterols, and primary bile acids may cause liver inflammation [16]. Hepatocellular carcinoma (HCC) cells are associated with cholesterol metabolism. HCC cells upregulate acetyl-CoA acetyltransferase (ACAT2) to remove oxysterols as bile acid precursors and avoid cell growth inhibition [17]. Therefore, the ratio of cholesterol to bile acid may be important in liver HCC pathology. Moreover, inflammation may be a crucial pathophysiological mechanism in HCC [18].

The regenerative pathway in response to damage to the liver includes activation of the inflammatory process and priming of hepatocytes to start a compensatory proliferative program aimed at controlling liver injury and restoring tissue homeostasis [19]. In the present study, we are showing for the first time that cholesterol intake protects against bile acid liver toxicity. We used two mouse models of bile acid toxicity with and without cholesterol exposure in order to induce liver inflammation and fibrosis. In both models, cholesterol administration had a hepatoprotective effect. Using a nutritional model of NASH (atherogenic diet including cholesterol and cholic acid) [20] and a genetic model of liver fibrosis (Mdr2-/- mice, ABCB4 KO mice), we showed that the hepatic cholesterol level regulates the disease phenotype. Cholesterol ameliorates bile acid toxicity, liver damage, and advanced fibrosis by activating the inflammatory/proliferative-regenerative pathways.

## 2. Materials and Methods

### 2.1. Animal Model and Treatment

#### 2.1.1. Diet-Induced NAFLD

Male C57BL/6J mice aged 6–7 wks were purchased from Harlan Laboratories, Jerusalem, Israel. Wild-type mice (*n* = 32) were randomly divided into four experimental groups: (1) standard AIN-93G diet (Control group), (2) standard AIN-93G diet+1% (*w*/*w*) cholesterol (CHOL group), (3) standard AIN-93G diet+0.5% (*w*/*w*) cholic acid (CA group), and (4) standard AIN-93G diet+1% (*w*/*w*) cholesterol+0.5% (*w*/*w*) cholic acid (CHOL+CA group). They were maintained on these regimens for 3 wks. Body weight and food consumption were recorded every 4 d.

#### 2.1.2. Fibrosis Study

Mdr2-/- (ABCB11) knockout mice were kindly provided by the laboratory of Prof. Eitan Galun, Ein-Kerem, The Hebrew University of Jerusalem. In all experiments comprising this study, female mice were used as they present with a more severe pathology than male mice. Female Mdr2-/- mice aged 6–7 wks (*n* = 14) were randomly divided into two experimental groups: (1) standard AIN-93G diet (Mdr2-/- Control group) and (2) standard AIN-93G diet+1% (*w*/*w*) cholesterol (Mdr2-/- CHOL group). They were maintained on these regimens for 6 wks. Body weight and food consumption were recorded every week.

All mice were maintained in temperature-controlled rooms under a 12 h light/dark cycle with *ad libitum* access to food and water. At the end of both experiments, the mice were fasted overnight and sacrificed by isoflurane (Piramal Critical Care Inc., Bethlehem, PA, USA) anesthesia. Liver tissue and blood samples were collected and stored at -80°C until use. All procedures were performed in accordance with the Institutional Animal Care and Use Committee (IACUC) of The Hebrew University of Jerusalem (Nos. AG-17-15357-3 and AG-16-14907-4).

Methods for evluation of blood parameters and biochemical analysis, liver histology and immunohistochemistry, RNA isolation and gene expression, and protein extraction and western blot analyses are all found in the supplementary materials.

### 2.2. Hepatic Growth Factor (HGF) Levels

HGF levels were assayed with a commercially available enzyme-linked immunosorbent assay (ELISA) kit (R&D Systems, Inc., Minneapolis, MN, USA) according to the manufacturer's instructions. In brief, liver tissues were homogenized in lysis buffer and centrifuged at 19,746 × *g* and 4°C for 15 min. Supernatants were collected and HGF concentrations were measured by colorimetry against a mouse/rat HGF standard.

### 2.3. Statistical Analysis

Significant differences between treatment means were identified by independent one-way ANOVA and a Tukey-Kramer test. Data are expressed as means ± SEM. *p* < 0.05 was considered statistically significant. JMP v. 13.0.0 (SAS Institute, Cary, NC, USA) was used for data processing.

## 3. Results

### 3.1. Weight and Food Intake

**Figure 1:**
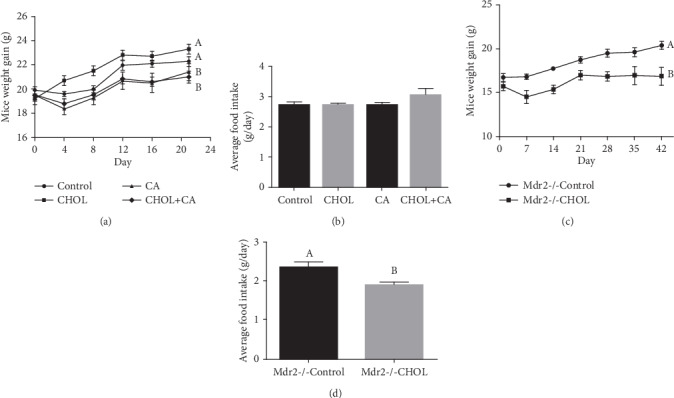
Body weight and food intake. (a) Body weight of wild-type mice supplemented with atherogenic diet constituents over experiment duration. (b) Average food intake of wild-type mice over experiment duration. All values are expressed as mean ± SEM (*n* = 7). (c) Body weight of Mdr2-/- mice over experiment duration. (d) Average food intake of Mdr2-/- mice over experiment duration. All values are expressed as mean ± SEM (*n* = 7). Means with different letters are statistically different, *p* < 0.05.

All wild-type mice were weighed every 4 d over the 21 d of the study. All four experimental groups presented with continuous weight gain (Figure 1(a)). The weight gains in the animals supplemented with cholic acid (CA and CHOL+CA groups) were smaller than that measured for the Control group. However, the food intake was similar for all groups (Figure 1(b)). The Mdr2-/- mice were weighed once weekly over the 42 d of the study. The Mdr2-/- Control group showed continuous weight gain (Figure 1(c)) and had a significantly higher weight than the Mdr2-/- CHOL group by the end of the experiment. The average food intake of the Mdr2-/- Control group was significantly higher than that of the Mdr2-/- CHOL group (Figure 1(d)). The clinical status of female Mdr2-/- mice was of active disease while the male mice were without clinical indications of cholestasis. The results are in agreement with previous reports, and the effect is related to different bile acid compositions and levels in the female compared to the male mice [21, 22]. In male mice, the effect of cholesterol was to exacerbate disease conditions as is shown in Figure 1 S. Since we sought to evaluate regenerative/therapeutic effects of cholesterol, we used the female mice for further analysis.

### 3.2. Liver Cholesterol Content and Physical Characteristics

**Figure 2:**
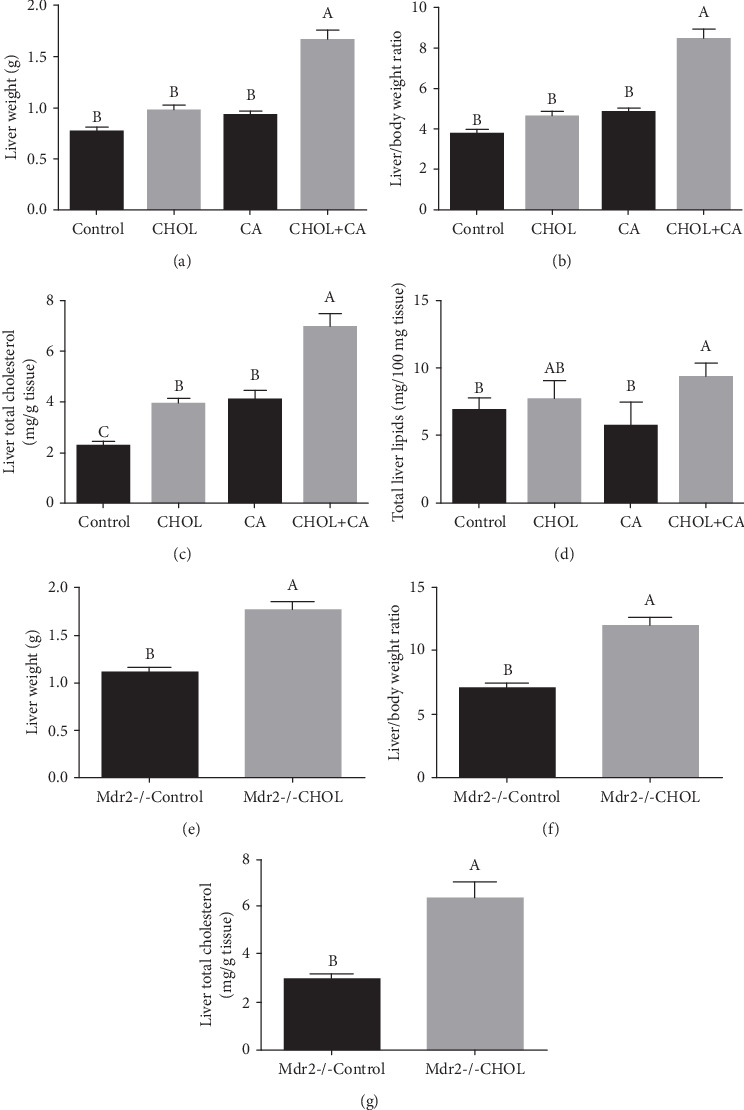
Liver weight and liver cholesterol content. (a) Liver weight at the end of the experiment of wild-type mice supplemented with atherogenic diet constituents. (b) Liver-to-body weight ratio of wild-type mice. (c) Hepatic cholesterol content of wild-type mice. (d) Hepatic lipid percentage of wild-type mice. All values are expressed as mean ± SEM (*n* = 8). (e) Liver weight at sacrifice of Mdr2-/- mice supplemented or not with cholesterol. (f) Liver-to-body weight ratio of Mdr2-/- mice. (g) Hepatic cholesterol content of Mdr2-/- mice. All values are expressed as mean ± SEM (*n* = 7). Means with different letters are statistically different, *p* < 0.05.

Relative to the Control, the liver weight markedly increased in response to supplementation with the atherogenic diet (Figures 2(a) and 2(b)). Cholesterol accumulation in wild-type mouse liver is shown in Figure 2(c). The Control group had significantly lower cholesterol levels than the other groups in the study. Groups provided with either cholic acid or cholesterol (CA and CHOL) had similar hepatic cholesterol levels. The CHOL+CA group had significantly (*p* < 0.05) higher cholesterol levels than the others in the study. The observed liver enlargement was not attributable to fat accumulation as the percentage of liver fat was essentially equal for all groups. Hepatic lipid accumulation is shown in Figure 2(d). Cholesterol-induced hepatomegaly was observed in the Mdr2-/- mice supplemented with cholesterol (Figures 2(e) and 2(f)). The hepatic cholesterol content was significantly higher in the group supplemented with it (Mdr2-/- CHOL) than the Mdr2-/- Control group (Figure 2(g)).

### 3.3. Serum Liver Damage Markers

**Figure 3:**
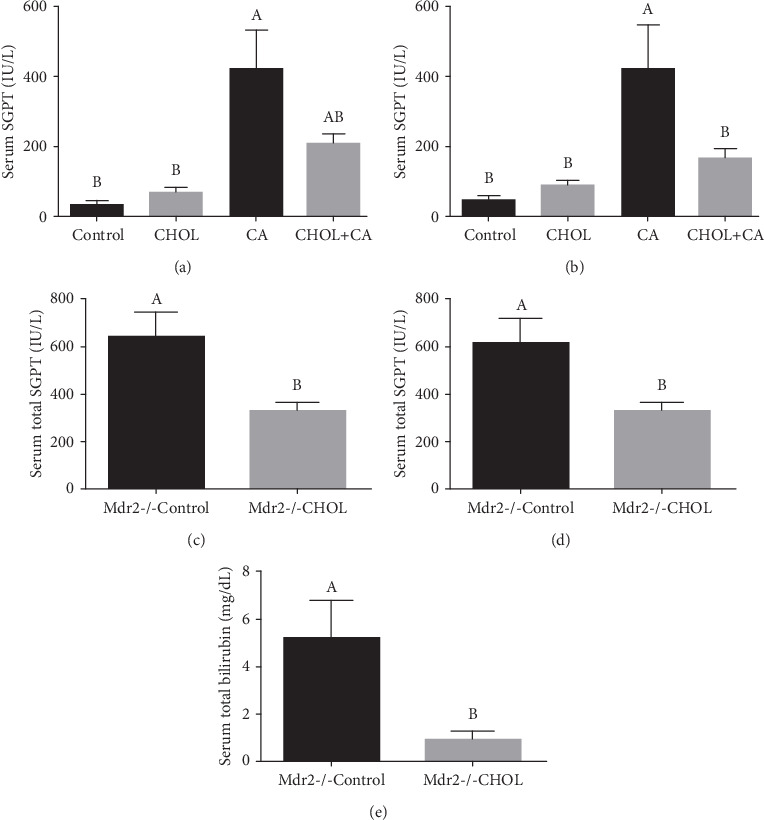
Hepatic blood liver enzymes and damage markers. Hepatic damage markers in serum of wild mice treated with atherogenic diet constituents: (a) serum SGPT levels and (b) serum SGOT levels. All values are expressed as mean ± SEM (*n* = 8). Hepatic damage markers in Mdr2-/- mice serum: (c) serum SGPT levels, (d) serum SGOT levels, and (e) serum bilirubin. All values are expressed as mean ± SEM (*n* = 7). Means with different letters are statistically different, *p* < 0.05.

Serum SGPT and SGOT upregulation was indicative of hepatic damage and hepatocyte destruction (Figure 3). In the wild-type mouse study, the CA group presented with significantly higher SGOT and SGPT levels than the others. There were no significant differences in liver enzyme level between the Control and CHOL groups (Figures 3(a) and 3(b)). The CHOL+CA group showed a significantly lower SGOT level than the CA group. The SGPT level was lower in the CHOL+CA group than in the CA group (*p* = 0.06). For the Mdr2-/- study, the serum hepatic damage markers such as bilirubin, SGOT, and SGPT were significantly lower in the treatment groups than in the Control group. Thus, cholesterol supplementation exerted a hepatoprotective effect (Figures 3(c), 3(d), and 3(e)). The inhibition of liver damage by cholesterol cannot be explained by suppressing bile acid production as cholesterol upregulated CYP7A1 and CYP27A1 which are the key enzymes for bile acid biosynthetic pathways (Figure 2 S).

### 3.4. Liver Histology and Fibrosis

**Figure 4:**
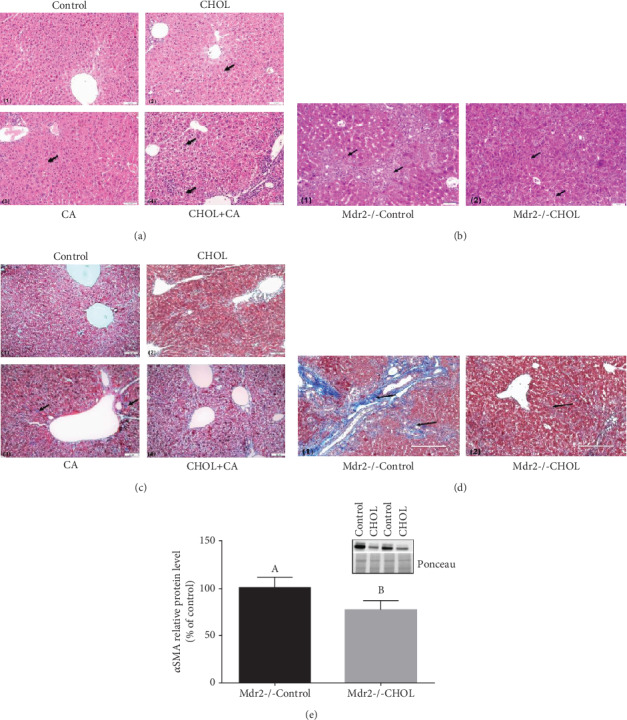
Liver histology and fibrosis. (a) Representative liver H&E staining of wild-type mice supplemented with atherogenic diet constituents. (1) Control liver sections. (2) Cholesterol- (CHOL-) treated group: presence of minor accumulation of lipid droplets. (3) CA-treated group: presence of necrotic cells with malformed nuclei. (4) CHOL+CA group: presence of ballooning degeneration of hepatocytes and accumulation of lipid droplets and Mallory-Denk bodies. Scale bar = 50 *μ*m. (b) Representative liver sections of H&E staining of Mdr2-/- mice supplemented or not with cholesterol. (1) Mdr2-/- Control group: presence of necrotic areas and cells with malformed nuclei. (2) Mdr2-/- CHOL group: presence of ballooning degeneration of hepatocytes, increased nuclear-cytoplasmic ratio, and Mallory-Denk bodies (right). Scale bar = 50 *μ*m. (c) Representative liver Masson's trichrome staining of wild-type mice. (1) Control group: no visible collagen lesions. (2) CHOL group: no visible collagen lesions. (3) CA group: presence of fibrotic collagen lesions colored blue. (4) CHOL+CA group: no visible collagen lesions. Scale bar = 50 *μ*m. (d) Representative liver Masson's trichrome staining of Mdr2-/- mice supplemented or not with cholesterol. (1) Mdr2-/- Control group: heavy blue staining of fibrotic collagen lesions. (2) Mdr2-/- CHOL group lightly stained lesions. Scale bar = 200 *μ*m. (e) Activation of fibrotic response: *α*SMA relative protein levels in liver tissue of Mdr2-/- mice, with and without cholesterol treatment. All values are expressed as mean ± SEM (*n* = 4). Means with different letters are statistically different, *p* < 0.05.

Hematoxylin and eosin (H&E) counterstaining of representative liver sections is shown in Figure 4. H&E staining of the livers of wild-type mice supplemented with atherogenic diet constituents and those of the Control (Figure 4(a)-1) and CHOL (Figure 4(a)-2) groups disclosed well-defined hepatocyte nuclei and normal hepatic-triad structure (Figure 4(a)). There was slight microvesicular fat accumulation in the CHOL group. The CA group (Figure 4(a)-3) presented with numerous damaged hepatocytes bearing malformed and shrunken nuclei. The CHOL+CA (Figure 4(a)-4) group showed increased nuclear-cytoplasmic ratios, nuclear hyperchromasia, microvesicular fat accumulation, major cellular infiltration in the periportal areas, hepatocyte ballooning, and Mallory-Denk hyaline.

In the Mdr2-/- study, the Control group (Figure 4(b)-1) presented with damaged hepatocytes displaying multiple malformed and necrotic nuclei and low nuclear counts. Nuclear staining appeared uneven. Whereas certain nuclei were lightly stained, others appeared dark and shriveled. Lightly stained lesions with multicellular infiltration and microvesicular fat accumulation were observed and stellate cell hyperplasia predominated. The Mdr2-/- CHOL group (Figure 4(b)-2) showed an increased nuclear-cytoplasmic ratio, nuclear hyperchromasia, and a higher nuclear count. The hepatocyte nuclei varied greatly in size and some cells were multinucleate. Overall, there was a dramatic reduction in the number of stellate cells. Mallory-Denk bodies and hepatocyte ballooning were detected throughout the tissue section.

Masson's trichrome staining was used to reveal hepatic collagen formation which is indicative of fibrosis and scarring. The development of scar tissue is a pathological hallmark of advanced liver damage. The damaged liver parenchyma is replaced by fibrous collagen (fibrosis). Comparison of liver tissue staining between unsupplemented and cholesterol-supplemented Mdr2-/- mice is shown in Figure 3 S. For the wild-type mice supplemented with atherogenic diet constituents, the Control group (Figure 4(c)-1) and cholesterol-supplemented groups CHOL (Figure 4(c)-2) and CHOL+CA (Figure 4(c)-4) showed little fibrous collagen accumulation. The CA (Figure 4(c)-3) group supplemented with cholic acid presented already after three weeks of diet some blue-staining liver tissue indicating fibrosis. Fibrous matter started accumulating from the periportal area and progressed to the deeper intralobular areas.

The influence of cholesterol on ameliorating liver fibrosis was very prominent in the Mdr2-/- mice (Figure 4(d)). The Mdr2-/- Control group (Figure 4(d)-1) demonstrated large numbers of fibrotic lesions in the periportal areas and slightly smaller yet important quantities of fiber formation in the intralobular areas (bright blue staining). In contrast, Mdr2-/- CHOL (Figure 4(d)-2) did not show substantial fibrous tissue formation but only presented with mild colorless lesions. Cholesterol supplementation significantly downregulated *α*SMA in the Mdr2-/- mice (Figure 4(e)).

### 3.5. Effects of Cholesterol on Liver Inflammation

**Figure 5:**
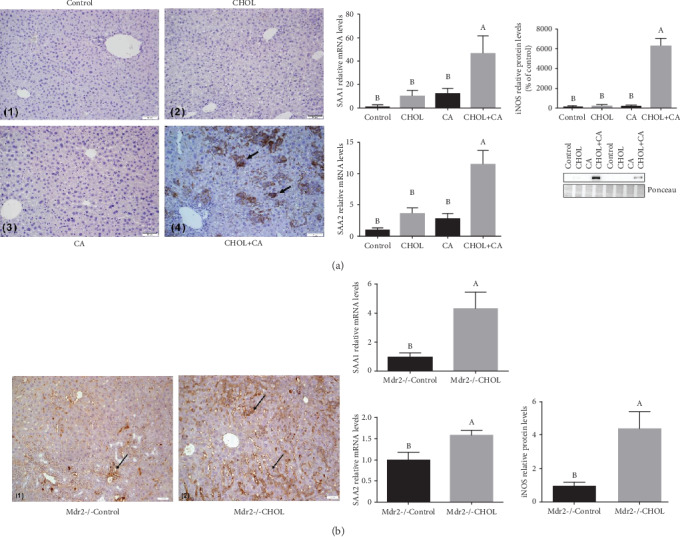
Inflammatory response in liver tissue. (a) IHC staining and analysis of inflammatory genes from liver tissue of wild-type mice supplemented with atherogenic diet constituents. IHC staining performed with antimacrophage marker F4/80. (1) Control group, (2) CHOL group, (3) CA group, and (4) CHOL+CA group: significant macrophage infiltration. Scale bar = 50 *μ*m. Gene expression: SAA1 and SAA2 relative mRNA levels and iNOS relative protein levels. All values are expressed as mean ± SEM (*n* = 8). (b) Inflammatory gene expression and IHC of liver tissue of Mdr2-/- mice performed with staining of macrophage marker F4/80. (1) Mdr2-/- Control group: brown areas stained with DAB represent slight macrophage infiltration. (2) Mdr2-/- CHOL group: major brown staining with DAB represents multiple, peripheral macrophage clusters. Scale bar = 50 *μ*m. Gene expression: SAA1, SAA2, and iNOS mRNA levels. All values are expressed as mean ± SEM (*n* = 7). Means with different letters are statistically different, *p* < 0.05.

Activation of the inflammatory process and acute phase response is the first indication for liver regenerative response to damage. Here, we evaluated the expression of multiple inflammatory markers (Figure 5) following bile acid-induced damage with and without cholesterol. F4/80 (M1 macrophage and activated Kuppfer cell marker) antibody and macrophage IHC staining were used to evaluate macrophage activation and infiltration. The CHOL+CA (Figure 5(a)-4) group liver tissue sections exhibited multiple areas that were distinctively stained brown by DAB. These regions are macrophage aggregation sites. The other treatment groups had substantially lower inflammatory responses. These results are correlated with the measured elevations in the levels of inflammatory response markers relevant to liver regeneration such as inducible nitric oxide synthase (iNOS) and serum amyloid A 1 and 2 (SAA1 and SAA2) mRNA and protein in the atherogenic diet-fed group (CHOL+CA (Figure 5(a)-4)).

Inflammatory markers and immunohistochemical staining for F4/80 in the liver sections of the Mdr2-/- mice are shown in Figure 5(b). IHC staining of the F4/80 was very much similar to that seen in the wild-type mouse study. The Mdr2-/- Control (Figure 5(b)-1) group exhibited small, diffuse brown areas which represented mild macrophage infiltration. In contrast, the Mdr2-/- CHOL (Figure 5(b)-2) group presented with considerable brown staining representing multiple peripheral macrophage clusters. Moreover, the mRNA levels of iNOS, SAA1, and SAA2 were significantly higher in the Mdr2-/- CHOL group than in the Mdr2-/- Control group (Figure 5(b)).

### 3.6. Hepatocyte Proliferation in Liver Tissue

**Figure 6:**
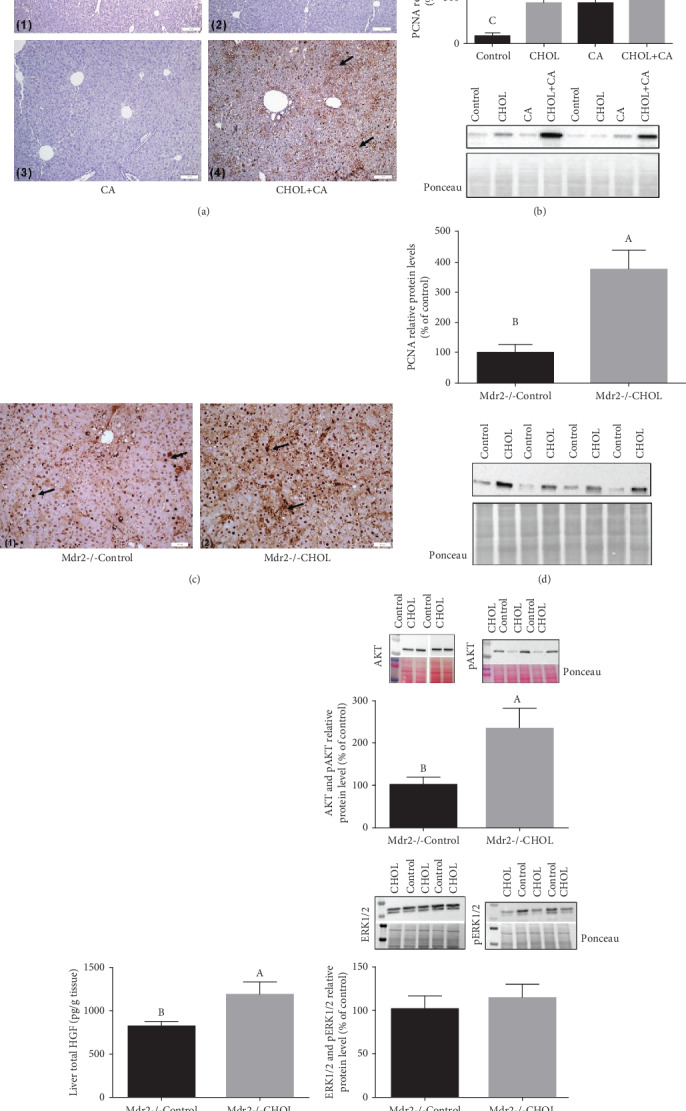
Proliferative effects of cholesterol in liver tissue. (a) IHC staining of liver tissue of wild-type mice supplemented with atherogenic diet constituents performed with PCNA staining: (1) Control group: no PCNA-positive nuclei. (2) CHOL group: no PCNA-positive nuclei. (3) CA group: no PCNA-positive nuclei. (4) CHOL+CA group: multiple brown-colored nuclei, stained with DAB, are nuclei of cells undergoing proliferative stage. Scale bar = 100 *μ*m. (b) PCNA relative protein levels in wild-type mice treated with atherogenic diet components. All values are expressed as mean ± SEM (*n* = 8). (c) IHC staining of liver tissue of Mdr2-/- mice performed with an anti-PCNA marker: (1) Mdr2-/- Control group: multiple brown-colored nuclei (DAB stained) of cells undergoing proliferative stage. (2) Mdr2-/- CHOL group: major brown staining (DAB staining) of multiple cell nuclei undergoing proliferative stage. Scale bar = 50 *μ*m. (d) PCNA relative protein levels in Mdr2-/- mice. (e) Hepatic growth factor levels (HGF) in Mdr2-/- mice. All values are expressed as mean ± SEM (*n* = 7). Means with different letters are statistically different, *p* < 0.05. (f) Increased phosphorylation of Akt and Erk proteins in Mdr2-/- mice treated with cholesterol for 6 weeks (*n* = 5), *p* < 0.05.

Hepatocyte multiplication was evaluated using proliferating cell nuclear antigen (PCNA), which is a DNA clamp that functions as a DNA polymerase processivity factor in eukaryotic cells. PCNA is essential for liver regeneration. The measured PCNA protein levels are shown in Figure 5. In correlation with increased liver cholesterol content (Figure 2(c)), PCNA protein was markedly upregulated in the CHOL+CA group relative to the other groups of the study in which wild-type mice were supplemented with atherogenic diet constituents (Figures 6(a) and 6(b)). The CHOL+CA group presented with abundant hepatocyte proliferation clusters (Figure 6(a)) near the portal triad. These mice also had significantly higher liver weights than the other groups in the study (Figure 2(a)). Other treatment groups of mice showed lower proliferative response and had statistically similar liver weights as control (Figure 2(a)).

Western blot and immunohistochemical staining for PCNA in representative liver sections of Mdr2-/- mice with or without cholesterol supplementation showed a trend similar to that for wild-type mice (Figures 6(c) and 6(d)). The Mdr2-/- CHOL had multiple, prominent, brown-stained nuclei expressing PCNA and comparatively higher liver weight. Hepatic growth factor (HGF) and PCNA protein were significantly upregulated in the Mdr2-/- CHOL group (Figure 6(e)). Cholesterol induces hepatocyte proliferation that correlates with activating the ERK1 and 2 and pAKT signaling pathways (Figure 6(f)).

We sought to evaluate the receptor tyrosine kinase MET and EGFR pathways for the activation of proliferative and regenerative response in the liver by cholesterol. By using the immunohistochemistry of pMET and pEGFR, it was shown that in the WT model of supplementation with atherogenic diet components, increased phosphorylation of both receptors was observed. In the MDR2KO models, the same proliferative effect of cholesterol was observed but a decrease in staining was observed (Figure 4 S). This indicates that the effect of cholesterol to activate proliferation is probably an intrinsic factor. Two such intrinsic factors that are related to proliferation and regeneration are hypoxia-inducible factor 1*α* (HIF-1*α*) and nuclear factor erythroid 2-related factor 2 (Nrf2).

No effect on liver progenitor of the cellular markers LGR5 and CK19 was observed following the cholesterol treatment (Figure 5 S).

### 3.7. HIF-1*α*-Mediated Response

**Figure 7:**
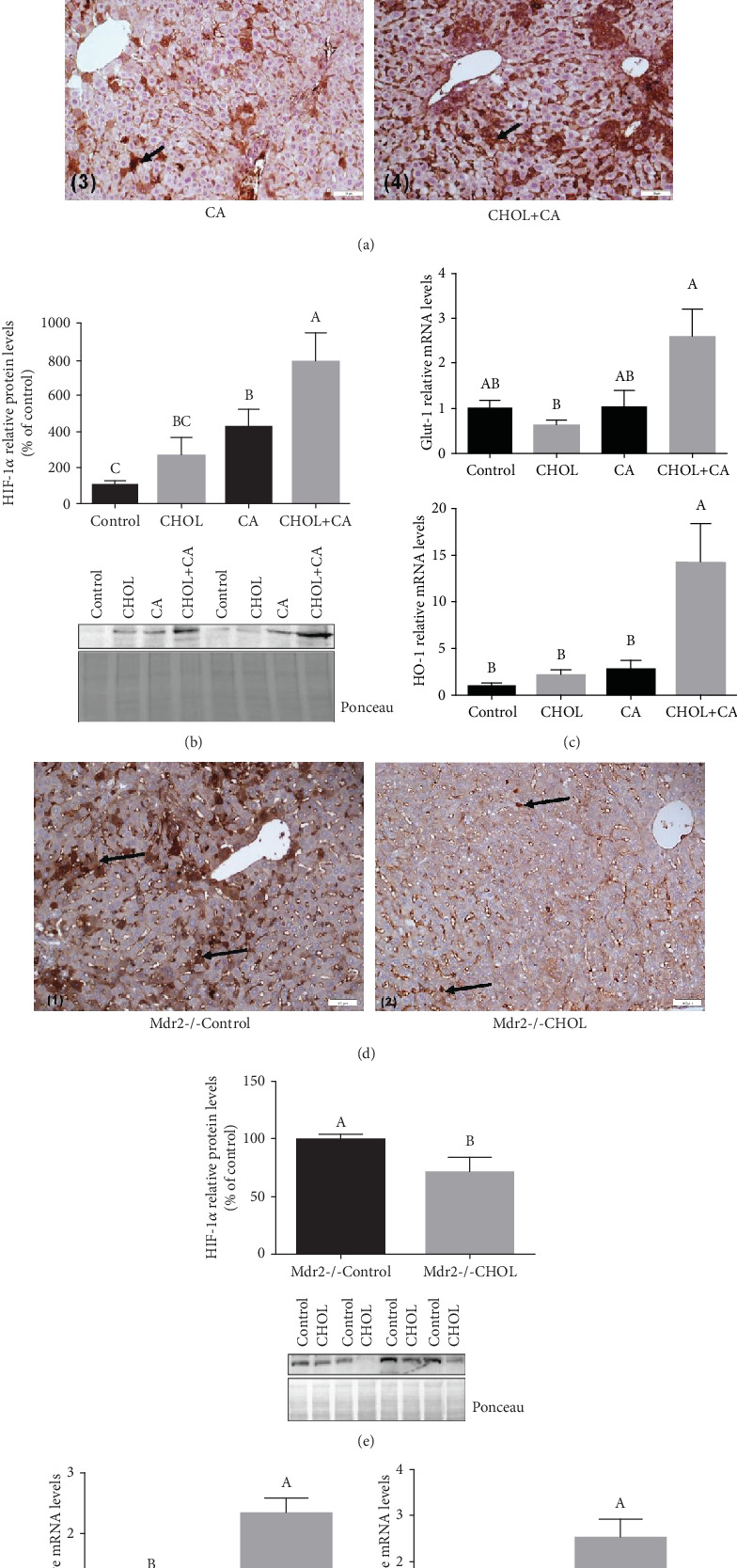
HIF-1*α*-mediated response. (a) IHC staining of liver tissue of wild-type mice treated with atherogenic diet constituents performed with anti-HIF-1*α* marker. (1) Control group: mild DAB staining of HSCs in extrahepatocyte area. (2) CHOL group and (3) CA group: prominent DAB staining of HSC aggregation areas. (4) CHOL+CA group: multiple clusters of hepatocyte-expressing HIF-1*α* marked with intense DAB staining. Scale bar = 50 *μ*m. (b) HIF-1*α* nuclear protein levels in hepatocytes of wild-type mice treated with atherogenic diet constituents. (c) HIF-1*α* downstream activity in hepatocytes of wild-type mice represented by Glut-1 and HO-1 relative mRNA levels. All values are expressed as mean ± SEM (*n* = 8). (d) IHC staining of liver tissue of Mdr2-/- mice performed with an anti-HIF-1*α* marker: (1) Mdr2-/- Control group: multiple brown-colored areas (DAB stained) of hepatic stellate cells (HSC) with elevated HIF-1*α* expression. (2) Mdr2-/- CHOL group: mild brown staining (DAB staining) of hepatic stellate cells (HSC). Scale bar = 50 *μ*m. (e) HIF-1*α* nuclear protein levels in hepatocytes of Mdr2-/- mice. (f) HIF-1*α* downstream activity in hepatocytes of Mdr2-/- mice represented by Glut-1 and HO-1 relative mRNA levels. All values are expressed as mean ± SEM (*n* = 7). Means with different letters are statistically different, *p* < 0.05.

The HIF-1*α* transcription factor mediates the adaptation of liver tissue to hypoxia and oxidative stress. Moreover, it is also associated with hepatocyte proliferation [23, 24] (Figure 7). HIF-1*α* protein expression levels were determined by IHC based on the HIF-1*α* antigen marked with DAB. In wild-type mice supplemented with atherogenic diet constituents (Figure 7(a)), the Control group mice presented with mild brown staining of the hepatic stellate cells (HSCs) in the extrahepatocellular areas. There was negligible staining of the hepatocyte nuclei. The CHOL and CA groups exhibited diffuse brown zones of HIF-1*α*-expressing HSCs. The CHOL+CA group showed elevated HIF-1*α* expression and prominent brown staining in numerous areas consisting of clusters of HSCs and hepatocytes. The CHOL+CA group also showed significant upregulation of the HIF-1*α* protein and its downstream genes Glut-1 and HO-1 (Figures 7(b) and 7(c)). The other groups (CHOL or CA) did not present with HIF-1*α* protein or HIF-1*α*-mediated gene upregulation. In this mouse model, there was minimal stellate cell hyperplasia and negligible fibrosis. When HIF-1*α* is expressed in stellate cells, it acts as a profibrotic factor [25]. In contrast, cholesterol had an antifibrotic effect in Mdr2-/- mice. Thus, HIF-1*α* and stellate cell hyperplasia were diminished and hepatocyte proliferation was augmented by cholesterol supplementation.

IHC staining with the HIF-1*α* antigen in the liver sections of Mdr2-/- mice is illustrated in Figure 7(d). In the Mdr2-/- Control group, the intensely colored brown areas are hepatic stellate cells (HSCs) with elevated HIF-1*α* expression. The Mdr2-/- CHOL group presented with only mild HIF-1*α* expression in the hepatocytes and stellate cells; its brown staining was diffuse compared to that of the Control. These findings are consistent with the measured HIF-1*α* protein expression levels (Figure 7(e)). HIF-1*α* expression was noted in the sinusoidal blood vessels of the liver reflecting vascular angiogenic response to cholesterol. According to the expression levels of the downstream genes Glut-1 and HO-1, HIF-1*α*-mediated activity was significantly elevated in the cholesterol-supplemented group (Mdr2-/- CHOL) (Figure 7(f)). In Mdr2-/- Control mice on the other hand, it was mainly hepatic stellate cells that expressed HIF-1*α*. These cells were practically eliminated following cholesterol supplementation.

### 3.8. Nrf-2-Mediated Antioxidant Response

**Figure 8:**
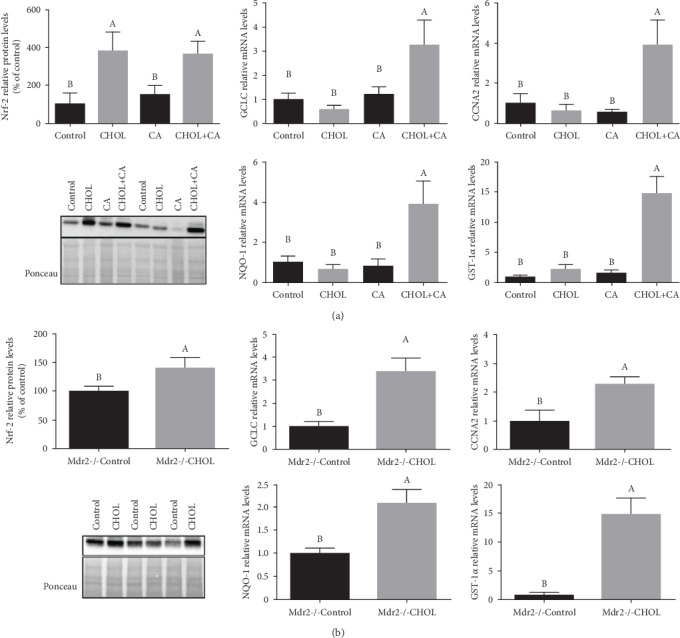
Nrf-2-mediated antioxidative response. (a) Nrf-2 nuclear protein levels and Nrf-2 downstream activity in wild-type mice supplemented with atherogenic diet constituents represented by HO-1 relative mRNA levels (Figure 7), GST-1*α* relative mRNA levels, GCLC relative mRNA levels, NQO-1 relative mRNA levels, and CCNA2 (cyclin A2) relative mRNA levels. All values are expressed as mean ± SEM (*n* = 8). (b) Nrf-2 nuclear protein levels and Nrf-2 downstream activity in Mdr2-/- mice treated or not with cholesterol. Nrf-2 activity is represented by HO-1 relative mRNA levels (Figure 7), GST-1*α* relative mRNA levels, GCLC relative mRNA levels, NQO-1 relative mRNA levels, and CCNA2 (cyclin A2) relative mRNA levels. All values are expressed as mean ± SEM (*n* = 7). Means with different letters are statistically different, *p* < 0.05.

The Nrf-2 transcription factor regulates the genes mediating liver responses to oxidative stress and is crucial for hepatocyte survival during liver regeneration [26]. For the wild-type mice supplemented with atherogenic diet constituents, the cholesterol-supplemented groups (CHOL and CHOL+CA) showed significant nuclear Nrf-2 protein upregulation relative to the Control and CA groups (Figure 8(a)). However, transcriptional upregulation of the Nrf-2-induced GCLC, GST-1*α*, and NQO-1 downstream genes was detected only in the atherogenic diet-fed group (CHOL+CA) indicating involvement of other transcription factors in the defense response (probably HIF-1*α*). Other groups receiving modified feeding regimes (CHOL and CA) did not statistically differ from the Control in terms of the expression levels of these genes.

The Nrf-2-mediated response to oxidative stress in the liver tissue was significantly higher for the Mdr2-/- CHOL group than the Mdr2-/- mice fed Control diet (Figure 8(b)). Nrf-2 nuclear protein level and GCLC, GST-1*α*, and NQO-1 mRNA levels were higher in the Mdr2-/- CHOL group than in the Mdr2-/- Control group. Cyclin A2 (*Ccna2*) levels, which connect Nrf-2 with the proliferative and regenerative response of the liver, were evaluated in both models. Cholesterol loading to the liver induced an elevation in Cyclin A2 (*Ccna2*) mRNA levels (Figure 8).

## 4. Discussion

Administration of cholesterol to mice subjected to liver bile acid toxicity resulted in damage correction. Cholesterol induced liver regeneration and activation of Nrf-2 and HIF-1*α* to increase hepatocyte protection against bile acids [26] and to induce hepatocyte proliferation. We demonstrated that cholesterol plays an important role in the disease trajectory to tissue fibrosis or cell proliferation. Several earlier studies suggested that cholesterol activates liver damage and fibrosis in NASH. However, in those reports only a small profibrotic effect was observed in response to the addition of cholesterol to a high-fat diet for 20 wks [27]. Another report stated that the fibrotic effect was not induced by cholesterol supplementation alone but rather the administration of profibrogenic agents after 4 wks of cholesterol supplementation was needed [27, 28]. Despite the common misperception, the progression of liver disease from steatosis to inflammation, fibrosis, and terminal HCC does not follow a linear relationship. Recent studies reported that around 50% of all NAFLD cases may progress to HCC without fibrosis [29, 30]. Therefore, hepatic fibrosis and hepatocyte or liver progenitor cell proliferation may be separate outcomes and counteracting effects rather than linked in a chain of interconnected events.

By separating the constituents of an atherogenic diet, our study showed that initial bile acid accumulation might be responsible for the liver damage and fibrosis. Liver damage was assessed in both models. Histological analysis disclosed that bile acids initiated fibrotic lesions in mice supplemented with cholic acids and induced bridging fibrosis in the MDR2-/- mice. These findings are consistent with serum liver enzyme elevation and are regarded as strong evidence for hepatic necrosis [31, 32]. Bile acids promote liver injury via their detergent and cytolytic action and by inducing endoplasmic reticulum stress and mitochondrial damage [33, 34]. Bile acids initiate the transdifferentiation of HSCs into myofibroblasts [35, 36] which ultimately leads to fibrosis. Bile acid toxicity leads to parenchymal necrosis and fibrosis and promotes the progression of cirrhosis as demonstrated by histological findings and serum liver enzyme levels [37]. Indeed, several studies emphasized the toxic effects of bile acids in NAFLD [38, 39].

Cholesterol supplementation with bile acids (CHOL+CA and Mdr2-/- CHOL groups) induced hepatic cholesterol accumulation, activated cell proliferation, and enlarged the liver. Threshold levels for cholesterol to activate inflammatory and regenerative response are achieved as cholesterol removal is inhibited by bile acids due to FXR activation [40]. The atherogenic diet caused a significant increase in liver mass; the livers of the CHOL+CA group were twice the size of those of the others. This effect was comparatively more dramatic in Mdr2-/- mice receiving dietary cholesterol. Groups receiving cholate had significantly lower triglyceride levels than the others (not shown), possibly also because of FXR activation leading to liver X receptor (LXR) inhibition [41, 42]. Evaluation of the bile acid profile in future studies could help to understand the metabolic fate of supplemented cholesterol and cholic acid.

### 4.1. Cholesterol Alleviates Bile Acid-Induced Damage by Liver Regeneration

Chronic liver inflammation was observed in the presence of cholesterol. In this immune response, inflammation, tissue remodeling, and repair occur simultaneously [43]. Hepatic cholesterol accumulation potently activates an inflammatory response in the liver. Cholesterol has been designated a proinflammatory molecule that can trigger steatohepatitis [44]. Upregulation of the biomarkers SAA1, SAA2, and iNOS revealed that cholesterol enrichment promoted profound inflammation [45, 46] involving hepatic innate immune cells (Kuppfer cells or KC). Activated KCs produce various proinflammatory cytokines such as SAA1 and SAA2 and recruit macrophages from systemic circulation into the liver tissue [47]. IHC staining for F4/80 characterized activated KCs and circulating M1 macrophages and displayed major cellular infiltration in the CHOL+CA and Mdr2-/- CHOL groups.

Circulatory macrophages are classified into “M1” and “M2” subsets [48]. The M1 macrophage phenotype is characterized by the production of matrix metalloproteinases- (MMP-) 2, 9, and 13 [49]. Experimental murine models demonstrated that M1 MMPs are essential for fibrolysis as they reduce extracellular matrix accumulation [48].

Liver regeneration following hepatotoxin-induced damage is fundamental in the response of the liver to injury. Here, we showed that concomitantly to the inflammatory response, cholesterol facilitates hepatocyte proliferation and tissue regeneration after bile acid injury. Cholesterol increased total AKT and ERK phosphorylation as markers of proliferation. In the Mdr2-/- model, nearly full recovery from liver fibrosis was demonstrated to be provoked by cholesterol administration. This phenomenon was concurrent with the activation of the hepatocyte proliferative response and phase 1 regeneration. Indeed, more severe damage that can activate liver progenitor cells was not observed (Figure 4 S). Thus, cholesterol affects the balance between hepatocyte proliferation/regeneration and liver tissue fibrosis in the attempt to restore organ homeostasis [50]. The activation of proliferation to ameliorate liver damage and lipotoxicity was demonstrated in a cMet KO mouse model indicating the capacity of increased proliferative response to offset liver damage [51]. Cholesterol is important for the process of liver regeneration and adequate proliferation due to its structural and membrane-related stabilizing properties. Thus, as cholesterol is the precursor of bile acids, increased bile acid synthesis can drain cholesterol levels, contributing to the negative effect of bile acids for the adequate proliferative response, suggesting that an appropriate cholesterol-to-bile acid ratio is required for hepatocyte proliferation and appropriate metabolism to ensure successful liver regeneration [52, 53].

### 4.2. Cholesterol Promotes the Induction of HIF-1*α* and Nrf-2 Defense Mechanisms

It was observed that treatment with atherogenic diet increases the pMET and pEGFR levels following cholesterol treatment but did not increase the phosphorylation in the Mdr2(-/-) mouse model (Figure 4 S). In both models, liver proliferation was observed. Therefore, the activation of proliferative response is probably an intrinsic effect of cholesterol in hepatocytes. In addition, successful regeneration is dependent on hepatocyte survival throughout the inflammatory process and requires induction of cellular survival and defense pathways [26]. Previously, it has been demonstrated that MiR-34a knockdown can significantly enhance the liver function and hepatocyte regeneration ability in rats at 10 d after hepatectomy through activating the Notch/HIF-1*α* signaling pathway similar to the effect of cholesterol loading to the liver. Increased liver/body weight ratio; a remarkable decline in serum levels of ALT, AST, and LDH; significant alleviation of pathological injury of liver tissues; and decreased apoptosis and upregulated PCNA protein were observed following partial hepatectomy [54]. The present study illustrated the various outcomes of HIF-1*α* induction in hepatocytes for antifibrotic and in HSCs for profibrotic response. Cholesterol-mediated HIF-1*α* activation in the hepatocytes was previously shown by our group and corroborates more recently reported findings [55]. Hepatocyte-expressed HIF-1*α* promotes adaptive mechanisms such as increased glucose transport and HO-1 to compensate for disrupted mitochondrial function and ROS elevation [56]. It was shown in an earlier study that HIF-1*α* and Nrf-2 activity induces HO-1 in hepatocytes and protects the liver from the fibrosis promoted by cholate supplementation [37]. HO-1 induction suppresses hepatic stellate cell activation and reverses fibrosis progression [21].

HIF-1*α* is probably associated with extrahepatocyte expression in the Mdr2-/- due to the fact that Glut-1 and HO-1 expression was not downregulated by cholesterol supplementation.

HIF-1*α* expression levels in HSC increase with fibrosis as shown in the Mdr2-/- mice receiving no cholesterol supplementation. Previous studies reported similar findings [57] and attributed a critical role in collagen deposition to HIF-1*α*. IHC staining of the Mdr2-/- Control group disclosed that HIF-1*α* is abundantly expressed in extrahepatocyte areas occupied mainly by HSCs. HIF-1*α* elevation in HSCs mediates their survival and may contribute to an increase in liver fibrosis. Moreover, HIF-1*α* expression induces the differentiation of hepatic stellate cells (HSCs) into myofibroblasts. These proliferate and migrate to injured areas where they secrete ECM and promote fibrosis [58]. In the present study, cholesterol supplementation dramatically downregulated HIF-1*α* in the extrahepatocyte area and stellate cells and reduced HSC hyperplasia.

Dietary cholesterol profoundly alters hepatic metabolism and regeneration. We explored the effects of a cholesterol-enriched diet on hepatic defense mechanisms activated by the Nrf-2 transcription factor. Since numerous observations suggested that cholesterol loading to hepatocytes activate oxidative stress, we postulated that it would activate also Nrf-2 signaling. Nrf-2 null mice exhibited delayed liver regrowth following partial hepatectomy. Nrf-2 is required for timely M phase entry of replicating hepatocytes by ensuring proper regulation of cyclin A2 and the Wee1/Cdc2/cyclin B1 pathway during liver regeneration [59]. Nrf-2 activates hepatic antioxidant defense mechanisms including the induction of antioxidant proteins and enzymes [60]. In both experimental models of the present study, cholesterol addition activated the Nrf-2 system. Elevated mRNA transcription of HO-1, GCLC, GST-1*α*, and NQO-1 measured in the present study were indicative of upregulated Nrf-2 activity. GCLC and GST-1*α* upregulation increased glutathione production and mitochondrial glutathione levels. Recent research showed that Nrf-2 is a major player in HCC. Elevated Nrf-2 expression is associated with HCC and other malignancies. Prolonged activation of Nrf-2 defense mechanisms could be detrimental as it might cause chemotherapy resistance in cancer cells [61]. Dysregulated Nrf-2 signaling promotes cellular proliferation, triggers vascularization, accentuates invasiveness, and confers drug resistance [62]. In the present study, both models displayed a significant induction of a hepatocyte proliferative response correlated with elevated Nrf-2 expression. In contrast, no malignancy-associated proliferation was observed here.

It is important to notice that in general, a female Mdr-/- mice model presents a much poorer clinical status than the male. This is probably the reason why the cholesterol proliferative effect following supplementation was beneficial and protective to ameliorate liver damage in females but not in males. In the male Mdr2-/- model, the clinical status of the liver was significantly better from the beginning. It is known that in patients, women suffering from NAFLD are more susceptible to liver complications (necroinflammation), while men are more susceptible to metabolic and cardiovascular impairment [63] indicating also gender differences.

More important is the nutritional model of WT animals, in which cholesterol was protecting against bile acid (CA) toxicity in WT mice. However, we are not claiming that eating extra cholesterol is beneficial for healthy individuals. Cholesterol could be a two-edged sword depending on many factors including the clinical situation.

In conclusion, the present study established that the balance between cholesterol and bile acid content in the liver determines disease trajectory towards necrosis, advanced fibrosis, and inhibited tissue regeneration. In our models, we are unable to predict the final outcome of the NASH disease.We suggest that the improvement of liver condition due to elevated regeneration will ameliorate fibrosis and prevent fulminant hepatic failure by temporary treatment with cholesterol. This may, however increase the risk for malignant processes that can lead to liver cancer. Cholesterol induces inflammation and the proliferation of hepatocytes and eliminates stellate cells in order to reverse fibrosis. Regulating the balance between cholesterol and bile acids may be of therapeutic use for liver disease.

## Data Availability

The corresponding author will make data available on request (oren.tirosh@mail.huji.ac.il).

## Abbreviations

NASH: Nonalcoholic steatohepatitis
NAFLD: Nonalcoholic fatty liver disease
FXR: Farnesoid X receptor
FC: Free cholesterol
HCC: Hepatocellular carcinoma
ACAT2: Acetyl-CoA acetyltransferase
HGF: Hepatic growth factor
iNOS: Inducible nitric oxide synthase
SAA1 and SAA2: Serum amyloid A 1 and 2
PCNA: Proliferating cell nuclear antigen
KC: Kuppfer cells
HIF-1*α*: Hypoxia-inducible factor 1*α*
Nrf-2: Nuclear factor erythroid 2-related factor 2
LXR: Liver X receptor
SGPT: Serum glutamic-pyruvic transaminase
SGOT: Serum glutamic-oxaloacetic transaminase.

## Supplementary Materials

Supplementary Materials Figure 1 S: hepatic liver damage markers in male Mdr2(-/-) mice after cholesterol supplementation for 6 weeks. (A) Hepatic damage markers in serum. Serum SGPT levels (left) and serum SGOT levels (right). All values are expressed as mean ± SEM (*n* = 5‐6). (B) Serum bilirubin levels (*n* = 5‐6). Means with different letters are statistically different, *p* < 0.05. (C) Liver fibrosis. Intralobular fibrosis is observed only after cholesterol supplementation (fibrosis: arrows). Figure 2 S: expression of bile acid-producing enzymes CYP7A1 and CYP27A1. (A) Analysis of mRNA from liver tissue of wild-type mice supplemented with atherogenic diet components (*n* = 6). (B) Gene expression of liver tissue from Mdr2-/- mice supplemented or not with cholesterol (*n* = 6). Figure 3 S: representative histology of liver for fibrosis staining (Masson's trichrome). Five animals from each treated group. Figure 4 S: representative histology of liver for pMET and pEGFR staining by immunohistochemistry. Five animals from each treated group. Figure 5 S: expression of liver progenitor cell markers following supplementation with atherogenic diet constituents (A) LGR5 and (B) CK19. Analysis of mRNA from liver tissue of wild-type mice supplemented with atherogenic diet components (*n* = 8).
