# Supplementary material for: Cholesterol Induces Nrf-2- and HIF-1α-Dependent Hepatocyte Proliferation and Liver Regeneration to Ameliorate Bile Acid Toxicity in Mouse Models of NASH and Fibrosis
Source: Oxid Med Cell Longev. 2020 May 25;2020:5393761. doi: 10.1155/2020/5393761 (PMC7271232; doi:10.1155/2020/5393761)
Supplement: Supplementary Materials — Figure 1 S: hepatic liver damage markers in male Mdr2(-/-) mice after cholesterol supplementation for 6 weeks. (A) Hepatic damage markers in serum. Serum SGPT levels (left) and serum SGOT levels (right). All values are expressed as mean ± SEM (n = 5‐6). (B) Serum bilirubin levels (n = 5‐6). Means with different letters are statistically different, p < 0.05. (C) Liver fibrosis. Intralobular fibrosis is observed only after cholesterol supplementation (fibrosis: arrows). Figure 2 S: expression of bile acid-producing enzymes CYP7A1 and CYP27A1. (A) Analysis of mRNA from liver tissue of wild-type mice supplemented with atherogenic diet components (n = 6). (B) Gene expression of liver tissue from Mdr2-/- mice supplemented or not with cholesterol (n = 6). Figure 3 S: representative histology of liver for fibrosis staining (Masson's trichrome). Five animals from each treated group. Figure 4 S: representative histology of liver for pMET and pEGFR staining by immunohistochemistry. Five animals from each treated group. Figure 5 S: expression of liver progenitor cell markers following supplementation with atherogenic diet constituents (A) LGR5 and (B) CK19. Analysis of mRNA from liver tissue of wild-type mice supplemented with atherogenic diet components (n = 8). [file 5393761.f1.docx]

***Supplementary methods***

***Blood parameters and biochemical analysis***

Blood was collected from the inferior vena cava at the end of the experiment. Plasma alanine aminotransferase (ALT/SGPT), aspartate aminotransferase (AST/SGOT), total cholesterol, high density lipoprotein (HDL), and triglycerides were measured by American Laboratories, Herzliya, Israel. Fasting glucose levels were measured in the tail-tip blood with a handheld Optimum Xceed glucometer (Abbott Diagnostic Care Ltd., Oxon, UK). Total lipid was extracted from the liver tissues by the Folch method [[1](#_ENREF_1)]. Cholesterol levels were determined with a quantification assay kit (Abcam, Cambridge, UK) according to the manufacturer’s protocol.

***Liver histology and immunohistochemistry***

Liver tissues were fixed in 4% formalin and embedded in paraffin molds. Liver sections were stained with hematoxylin-eosin (H&E) for histological assessment according to standard methods. This procedure was performed by L.E.M Laboratories, Ness Ziona, Israel. Immunohistochemistry was performed using these antibodies: anti-proliferating cell nuclear antigen (PCNA), anti-F4/80 (Abcam, Cambridge, UK), and anti-HIF-1α (Novus Biologicals, Centennial, CO, USA), anti Phospho-Met (Tyr1234/1235) (D26) XP® Rabbit mAb (cell signaling) and

Anti Phospho-EGF Receptor (Tyr1068) (D7A5) XP® Rabbit mAb (cell signaling). Fibrosis staining was performed with a Masson trichrome staining kit (Bio-Optica, Milan, Italy) according to the manufacturer’s instructions.

***RNA isolation and gene expression***

Total RNA was isolated by the Tri-Reagent (Sigma-Aldrich, Jerusalem, Israel) method according to the manufacturer's protocol. Complementary DNA (cDNA) was prepared with a high-capacity cDNA reverse transcription kit (Quanta Biosciences, Beverly, MA, USA). Real-time PCR was performed in a 7300 Real-Time PCR System (Applied Biosystems, Warrington, UK) using specific primers (see the Supplementary Information). Fold changes in gene expression were determined by normalization to 18S mRNA.

***Protein extraction and western blot analyses***

Whole-cell lysates were prepared in lysis buffer. Nuclear extracts were prepared as previously described [[2](#_ENREF_2)]. Protein aliquots were subjected to western blot analysis. Ponceau S (Sigma-Aldrich, Jerusalem, Israel) staining was used to verify equal loading and transfer. Western blot analysis was performed by chemiluminescence detection (Bio-Rad Laboratories, Hercules, CA, USA) and densitometry. The following primary antibodies were used: anti-PCNA (Abcam, Cambridge, UK), anti-iNOS (BD Biosciences, Franklin Lakes, NJ, USA), anti-Nrf-2 (Santa Cruz Biotechnology, Dallas, TX, USA), anti-HIF-1α (Novus Biologicals, Centennial, CO, USA), anti- α-smooth muscle actin (Abcam, Cambridge, UK) [1A4] (ab7817), anti-ERK1/2 (9101, Cell-signaling technology. Inc) and anti-pERK1/2 (9102, Cell-signaling technology. Inc), anti-AKT(9272, Cell-signaling technology. Inc) and anti-pAKT(9271, Cell-signaling technology. Inc). Total protein count, obtained from ponceau staining, served as the reference standard for all calculations and band normalization. Secondary antibodies were purchased from Jackson ImmunoResearch, West Grove, PA, USA.

[1] Folch J, Lees M, Sloane Stanley GH. A simple method for the isolation and purification of total lipides from animal tissues. The Journal of biological chemistry 1957;226:497-509.

[2] Carey MF, Peterson CL, Smale ST. Dignam and Roeder nuclear extract preparation. Cold Spring Harbor protocols 2009;2009:pdb prot5330.

**Supplementary figure legends**

**Figure 1 S**. Hepatic liver damage markers in male Mdr2(-/-) mice after cholesterol supplementation for 6 weeks. (A) Hepatic damage markers in serum. Serum SGPT levels (left) and serum SGOT levels (right). All values are expressed as mean ± SEM (n=5-6). (B) Serum bilirubin levels (n=5-6). Means with different letters are statistically different, p<0.05. (C) Liver fibrosis. Intralobular fibrosis is observed only after cholesterol supplementation (fibrosis: arrows).

**Figure 2 S**. Expression of bile acid producing enzyme CYP7A1 and CYP27A1. (A) analysis of mRNA of liver tissue of wild type mice supplemented with atherogenic diet components n=6. (B) Gene expression liver tissue of Mdr2-/- mice supplemented or not with cholesterol n=6.

**Figure 3 S**. representative histology of liver for fibrosis staining (masson tricrome). 5 animals of each treated group.

**Figure 4 S**. representative histology of liver for pMET and pEGFR staining by immunohistochemistry. 5 animals of each treated group.

**Figure 5 S**. Expression of liver progenitor cell markers following supplementation with atherogenic diet constituents (A) LGR5 and (B) CK19. Analysis of mRNA of liver tissue of wild type mice supplemented with atherogenic diet components (n=8).
